# Aspergillus fumigatus Transcription Factors Involved in the Caspofungin Paradoxical Effect

**DOI:** 10.1128/mBio.00816-20

**Published:** 2020-06-16

**Authors:** Clara Valero, Ana Cristina Colabardini, Jéssica Chiaratto, Lakhansing Pardeshi, Patrícia Alves de Castro, Jaire Alves Ferreira Filho, Lilian Pereira Silva, Marina Campos Rocha, Iran Malavazi, Jonas Henrique Costa, Taícia Fill, Mário Henrique Barros, Sarah Sze Wah Wong, Vishukumar Aimanianda, Koon Ho Wong, Gustavo H. Goldman

**Affiliations:** a Faculdade de Ciências Farmacêuticas de Ribeirão Preto, Universidade de São Paulo Ribeirão Preto, Ribeirão Preto, Brazil; b Faculty of Health Sciences, University of Macau, Macau SAR, China; c Genomics and Bioinformatics Core, Faculty of Health Sciences, University of Macau, Macau SAR, China; d Departamento de Genética e Evolução, Centro de Ciências Biológicas e da Saúde, Universidade Federal de São Carlos, São Carlos, São Paulo, Brazil; e Instituto de Química, Universidade de Campinas, Campinas, São Paulo, Brazil; f Departamento de Microbiologia, Instituto de Ciências Biomédicas, Universidade de São Paulo, São Paulo, Brazil; g Unité Mycologie Moléculaire, Institut Pasteur, UMR2000, CNRS, Paris, France; h Institute of Translational Medicine, University of Macau, Macau SAR, China; Duke University Medical Center

**Keywords:** *Aspergillus fumigatus*, calcium, caspofungin, cell wall, transcription factors, mitochondria

## Abstract

Aspergillus fumigatus is the leading cause of pulmonary fungal diseases. Azoles have been used for many years as the main antifungal agents to treat and prevent invasive aspergillosis. However, in the last 10 years there have been several reports of azole resistance in A. fumigatus and new strategies are needed to combat invasive aspergillosis. Caspofungin is effective against other human-pathogenic fungal species, but it is fungistatic only against A. fumigatus. Resistance to caspofungin in A. fumigatus has been linked to mutations in the *fksA* gene that encodes the target enzyme of the drug β-1,3-glucan synthase. However, tolerance of high caspofungin concentrations, a phenomenon known as the caspofungin paradoxical effect (CPE), is also important for subsequent adaptation and drug resistance evolution. Here, we identified and characterized the transcription factors involved in the response to CPE by screening an A. fumigatus library of 484 null transcription factors (TFs) in CPE drug concentrations. We identified 11 TFs that had reduced CPE and that encoded proteins involved in the basal modulation of the RNA polymerase II initiation sites, calcium metabolism, and cell wall remodeling. One of these TFs, FhdA, was important for mitochondrial respiratory function and iron metabolism. The Δ*fhdA* mutant showed decreased growth when exposed to Congo red or to high temperature. Transcriptome sequencing (RNA-seq) analysis and further experimental validation indicated that the Δ*fhdA* mutant showed diminished respiratory capacity, probably affecting several pathways related to the caspofungin tolerance and resistance. Our results provide the foundation to understand signaling pathways that are important for caspofungin tolerance and resistance.

## Abstract (precis)

Aspergillus fumigatus, one of the most important human-pathogenic fungal species, is able to cause aspergillosis, a heterogeneous group of diseases that presents a wide range of clinical manifestations. Invasive pulmonary aspergillosis is the most serious pathology in terms of patient outcome and treatment, with a high mortality rate ranging from 50% to 95% primarily affecting immunocompromised patients. Azoles have been used for many years as the main antifungal agents to treat and prevent invasive aspergillosis. However, there were several reports of evolution of clinical azole resistance in the last decade. Caspofungin, a noncompetitive β-1,3-glucan synthase inhibitor, has been used against A. fumigatus, but it is fungistatic and is recommended as second-line therapy for invasive aspergillosis. More information about caspofungin tolerance and resistance is necessary in order to refine antifungal strategies that target the fungal cell wall. Here, we screened a transcription factor (TF) deletion library for TFs that can mediate caspofungin tolerance and resistance. We have identified 11 TFs that are important for caspofungin sensitivity and/or for the caspofungin paradoxical effect (CPE). These TFs encode proteins involved in the basal modulation of the RNA polymerase II initiation sites, calcium metabolism or cell wall remodeling, and mitochondrial respiratory function. The study of those genes regulated by TFs identified in this work will provide a better understanding of the signaling pathways that are important for caspofungin tolerance and resistance.

## INTRODUCTION

Infections by human-pathogenic fungal species are a major cause of morbidity and mortality in immunosuppressed patients. It is estimated that more people die from the 10 main invasive fungal diseases than from tuberculosis or malaria. Among them, invasive aspergillosis is the most common human systemic infection caused by filamentous fungi, with approximately 200,000 infections by year worldwide and a high rate of mortality that can reach 95% (1). Despite of the continuous increase of populations susceptible to fungal infections, the availability of antifungal drugs is still very limited. Azoles have been used for many years as the main antifungal agents to treat and prevent invasive aspergillosis (2, 3). Their mode of action relies on inhibition of ergosterol biosynthesis, which, coupled with the accumulation of a toxic intermediate, disrupts cell membrane integrity (4). However, in recent decades, a steady increase in worldwide reports of azole resistance in Aspergillus fumigatus, resulting in therapeutic failures, has been a matter of serious clinical concern (5). Resistant phenotypes have been found to be associated with exposure to azole formulations during both antifungal therapy in patients and fungicide application in the environment (6).

Due to the limited availability of effective antifungal agents and the emergence of azole resistance, new strategies are needed to combat invasive aspergillosis (7). Since fungal cell wall is absent in humans and is essential to fungal viability, drugs disrupting cell wall biosynthesis have gained more attention. Echinocandins, such as caspofungin (CSP), are the newest class of antifungal drugs approved to treat invasive fungal infections. They target the fungal cell wall by inhibiting 1,3-β-d-glucan synthase, which is responsible for the assembly of β-d-glucan, a major component of the fungal cell wall (8). As a result, treatment with echinocandins leads to hyphae with defects in growth and morphology (9). These antifungal agents are also effective against *Candida* species, exhibiting fungicidal activity. In contrast, they are fungistatic against A. fumigatus, promoting the lysis of the apical tips of expanding hyphae, altering hyphal morphology, and modifying cell wall composition and organization (10). Regardless, they are recommended for second-line therapy against invasive aspergillosis due to excellent tolerability and synergistic potential in combination with other antifungals (11). Resistance to echinocandins in A. fumigatus has been linked to mutations in the *fksA* gene (coding for 1,3-β-d-glucan synthase, the drug-target), representing a well-documented mechanism in *Candida* species (12, 13). Nevertheless, FKS1-independent resistance mechanisms have also been reported to exist (14, 15). A phenomenon different from resistance is known to occur when A. fumigatus is exposed to high concentrations of caspofungin, called the caspofungin paradoxical effect (CPE), which relies on the ability of the fungus to restore growth at suprainhibitory drug concentrations (16). Several studies have been performed to elucidate the mechanisms underlying this process, showing links between CPE and components of signaling pathways responsible for the fungal response to environmental stresses such as those imposed by Hsp90, calcineurin, or mitogen-activated protein kinases (MAPKs) (17–19). In Candida albicans, aneuploidy also enables the CPE by altering the copy number of several genes involved in cell wall remodeling or calcium metabolism (20, 21). Recently, our group identified a novel basic leucine zipper ZipD transcription factor (TF) that was found to play a role in the calcium-calcineurin pathway and to be involved in CPE (22, 23). Moreover, it has been suggested that reactivation of β-1,3-glucan synthase is essential for CPE maintenance (24).

Given the polygenic and complex nature of CPE, a better understanding of mechanisms of echinocandin resistance and tolerance in A. fumigatus has become mandatory. Accordingly, in order to identify possible regulatory mechanisms controlling CPE, we screened an A. fumigatus library of null TFs in various CPE concentrations. In this work, we identified and characterized several TFs involved in the response to CPE. We identified 11 TF mutants that have reduced CPE, and 9 of them were more sensitive to caspofungin than the wild-type strain. The TFs identified encode proteins involved in the basal modulation of the RNA polymerase (Pol) II initiation sites, in calcium metabolism, or in cell wall remodeling. Additionally, one of these TFs (FhdA) encodes a novel protein important for mitochondrial respiratory function and iron metabolism.

## RESULTS

### Eleven transcriptional factors govern caspofungin paradoxical effect (CPE).

**FIG 1:**
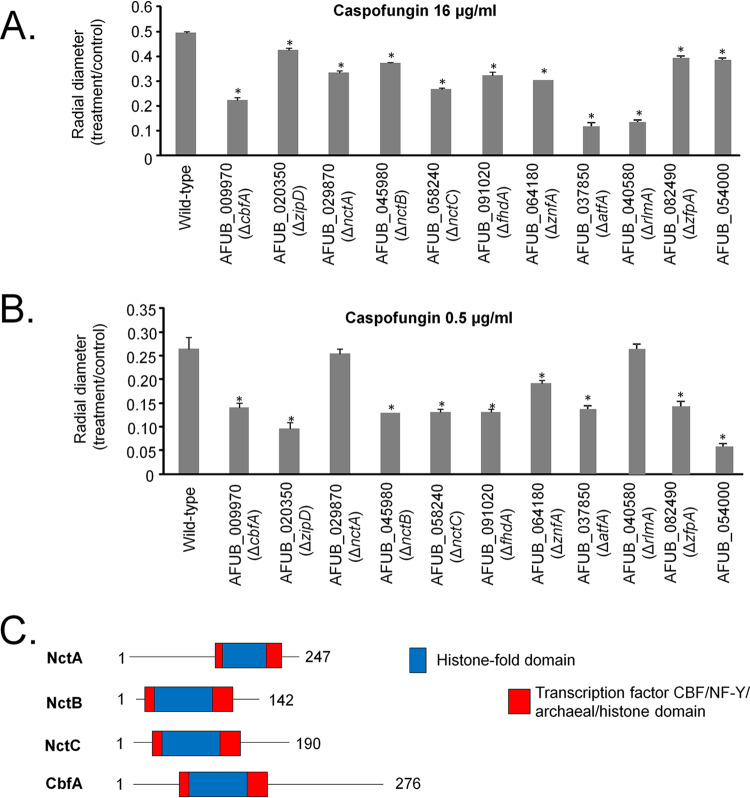
Identification of A. fumigatus transcription factor null mutants that have reduced caspofungin paradoxical effect (CPE). A collection of A. fumigatus 484 null transcription factor mutants was screened for sensitivity to caspofungin by growing them on MM plus 16 μg/ml of caspofungin. (A and B) Eleven mutants that displayed reduced CPE had their radial growth quantified on MM plus 16 μg/ml of caspofungin (A) or MM plus 0.5 μg/ml of caspofungin (B). Statistical analysis was performed using one-tailed, paired *t* tests for comparisons to the control condition (*, *P* < 0.05). (C) Protein domains in NctA, NctB, NctC, and CbfA determined by the use of SMART (http://smart.embl-heidelberg.de/).

To assess if any other TF plays a role in CPE, a library of 484 A. fumigatus TF null mutants (25) was screened for sensitivity to high concentrations of caspofungin (16 μg/ml). At this concentration, the CPE is induced in the CEA17 parental strain (22, 23). Eleven null mutants, including the Δ*zipD* mutant (ΔAFUB_020350), exhibited increased sensitivity to caspofungin at a concentration of 16 μg/ml (the other 10 mutants included ΔAFUB_009970 [*cbfA*], ΔAFUB_029870 [*nctA*], ΔAFUB_045980 [*nctB*], ΔAFUB_058240 [*nctC*], ΔAFUB_091020 [*fhdA*], ΔAFUB_064180 [*znfA*], ΔAFUB_037850 [*atfA*], ΔAFUB_040580 [*rlmA*], ΔAFUB_08249 [*zfpA*], and ΔAFUB_054000) (Fig. 1A). Except for the Δ*nctA* and Δ*rlmA* mutants, all also showed sensitivity to lower caspofungin concentrations, suggesting a high correlation between caspofungin sensitivity and reduced CPE (Fig. 1B). The Δ*nctA* and Δ*nctB* mutant strains were previously described (25) in a report from a study whose results indicated that loss of the negative cofactor 2 complex led to resistance to most clinically used antifungals, including azoles and terbinafine. We have confirmed these results for the Δ*nctA* mutant, but there were no differences in antifungal drug susceptibility seen for any other TF mutant in comparison with the wild-type strain (Table 1).

**TABLE 1:** MIC values and ranges obtained by CLSI antifungal susceptibility testing method for the A. fumigatus mutant strains identified in the caspofungin screening and the corresponding wild-type strain

Strain	MIC (μg/ml)^*a*^			
	Amphotericin B	Voriconazole	Itraconazole	Posaconazole
Wild-type A. fumigatus	2	0.25	0.5–1	1
A. fumigatus Δ*cbfA*	2	0.5	0.5	1
A. fumigatus Δ*zipD*	2	0.25	0.5	1
A. fumigatus Δ*nctA*	2	2	>16	4
A. fumigatus Δ*nctB*	2	0.5	0.5	1
A. fumigatus Δ*nctC*	2	0.25	0.25	1
A. fumigatus Δ*fhdA*	2	0.25	0.5	1
A. fumigatus Δ*znfA*	2	0.25–0.5	0.5	1
A. fumigatus Δ*atfA*	2	0.25	0.5	1
A. fumigatus Δ*rlmA*	2	0.5	1.0	1
A. fumigatus Δ*zfpA*	2	0.5	0.25–0.5	1
A. fumigatus ΔAFUB_054000	2	0.25–0.5	0.25	1

a Reference MIC values for A. fumigatus ATCC 204305 (μg/ml): amphotericin B, 0.5 to 2; voriconazole, 0.5 to 4; itraconazole, 0.12 to 1; posaconazole, 0.06 to 0.5.

CbfA is the putative homologue of Saccharomyces cerevisiae DPB4, a putative subunit of the DNA polymerase epsilon and the ISW2 chromatin accessibility complex (identity, 34%; similarity, 55%; E value, 4e−09). NctA and NctB, together with CbfA and NtcC, are members of the evolutionarily conserved CBF/NF-Y family of TFs (Fig. 1C; Pfam PF00808, histone-like transcription factor CBF/NF-Y and archaeal histone). This family encompasses archaebacterial histones and histone-like TFs from eukaryotes, which include the CBC transcription regulator complex (26). NctC is a putative A. fumigatus paralogue of the NctA and HapE CCAAT-binding factor complex subunit (26). FhdA and ZnfA encode novel TFs with fork-head-associated domains and zinc-finger-associated domains, respectively. AtfA is important for viability of conidia, and several kinds of stress, including oxidative, osmotic, and cell wall damage stress, while RlmA is required for the regulation of the cell wall integrity (CWI) pathway; both proteins have already been shown to be important for CPE (23, 27–30). We also previously found ZfpA transcripts upregulated in the presence of calcium and voriconazole (31, 32). However, AFUB_054000 is not known to have identity with any other known TF.

To obtain insight into the TF signaling networks governing caspofungin tolerance, we generate functional gene networks using STRING (https://string-db.org/) analysis. The TF interaction network showed two distinct networks that are highly connected and composed as follows: (i) CbfA (AFUB_009970), NctA (AFUB_029870), NctB (AFUB_045980), and NctC (AFUB_058240) and (ii) AtfA (AFUB_037850) and RlmA (AFUB_040580) (data not shown). These results suggest that the different TF members of the CBF/NF-Y family interact under conditions of caspofungin tolerance. In addition, RlmA and AtfA interact for cell wall remodeling under conditions of caspofungin tolerance.

Here, we characterized in more detail the Δ*cbfA*, Δ*nctC*, and Δ*fhdA* mutants.

### CbfA and NctC are required for optimal growth under conditions of stresses and suppress production of secondary metabolites (SM).

**FIG 2:**
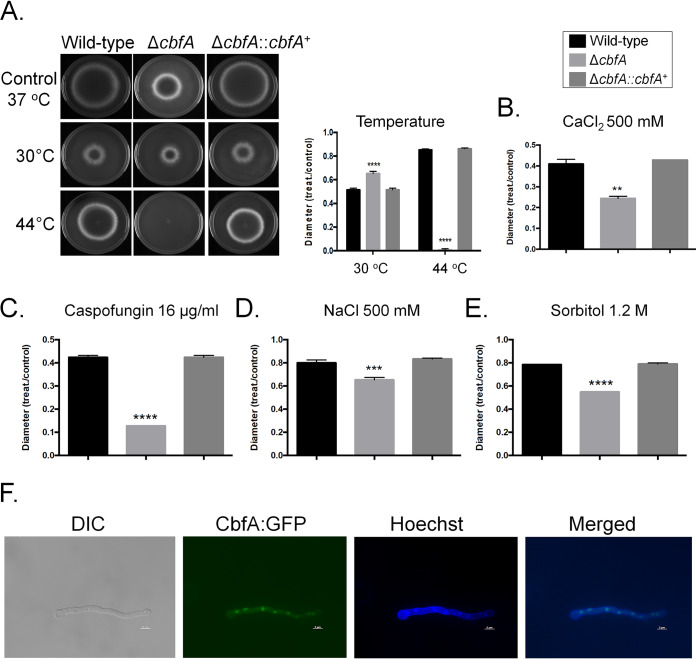
Molecular characterization of CbfA. Growth phenotypes of the wild-type, Δ*cbfA*, and Δ*cbfA*::*cbfA*^+^ strains grown for 5 days in MM at different temperatures (A), in MM plus CaCl_2_ 500 mM at 37°C (B), in MM plus 16 μg/ml of caspofungin at 37°C (C), in MM plus NaCl 500 mM at 37°C (D), and in MM plus sorbitol 1.2 M (E). Statistical analysis was performed using one-tailed, paired *t* tests for comparisons to the control condition (*, *P* < 0.05; **, *P* < 0.01; ***, *P* < 0.001; ****, *P* < 0.0001). (F) CbfA:GFP germlings (16 h old) were constitutively present in the nuclei.

**FIG 3:**
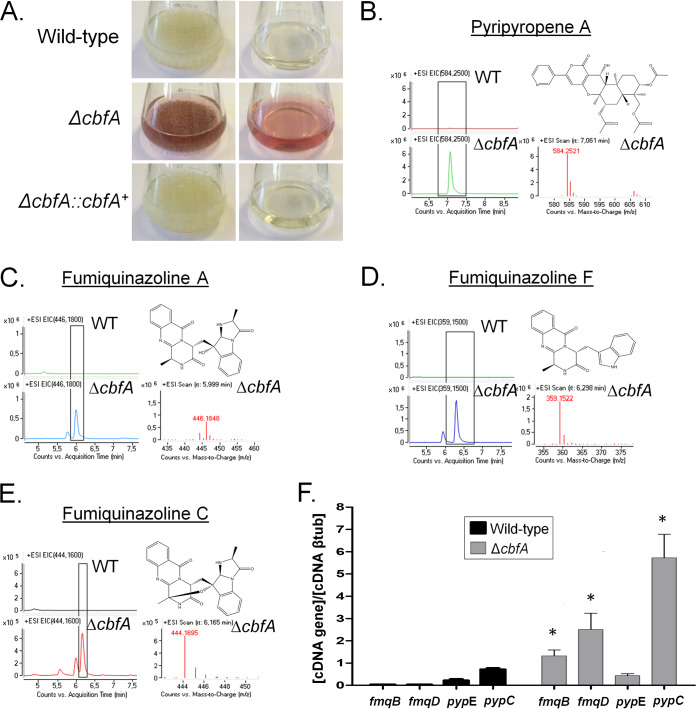
The Δ*cbfA* mutant has increased production of several secondary metabolites. (A) Cultures (left column) and supernatants separated from the same cultures (right column) were grown with agitation for 5 days at 37°C. (B to E) Secondary metabolites were extracted from the wild-type and Δ*cbfA* cultures from those supernatants, and mass spectrometry was performed, identifying pyripyropene A (B), fumiquinazoline A (C), fumiquinazoline F (D), and fumiquinazoline C (E). WT, wild type; EIC, extracted ion chromatogram. (F) Determination of the expression of *fmqB*, *fmqD*, *pypE*, and *pypC* by RT-qPCR. βtub, β-tubulin. The wild-type and Δ*cbfA* strains were grown for 48 h at 37°C. Gene expression was normalized using tubA (Afu1g10910). Standard deviations represent averages of results from three independent biological repetitions. Statistical analysis was performed using one-way ANOVA for comparisons to the wild-type condition (*, *P* < 0.05).

The Δ*cbfA* mutant showed reduced radial growth on minimal medium (MM) (about 50% of the levels seen with the wild-type and complemented strains; Fig. 2A), and it was more sensitive to higher temperature (44°C), CaCl_2_, caspofungin, and increased osmotic pressure (Fig. 2A to E). CbfA:GFP (CbfA:green fluorescent protein) was constitutively present in the nucleus (Fig. 2F) since there was no change in its location or in the level of fluorescence intensity seen upon exposure to all stressing conditions described above (data not shown). Interestingly, growth of the Δ*cbfA* mutant in liquid MM for up to 48 h at 37°C resulted in the secretion of compounds that gave a reddish color to the culture medium (Fig. 3A). Mass spectrometry analysis revealed that the Δ*cbfA* mutant showed increased production of fumiquinazoline A, C, and F and of pyripyropene A (Fig. 3B to E). Reverse transcription-quantitative PCR (RT-qPCR) confirmed that two genes in the fumaquinazoline pathway, *fmqB* (AFUB_078050) and *fmqD* (AFUB_047560), and one in the pyripyropene A biosynthesis pathway, *pypC* (AFUB_081560), were upregulated by about 2-fold to 10-fold in the Δ*cbfA* strain (Fig. 3F), suggesting that CbfA has a negative role in these SM gene clusters.

**FIG 4:**
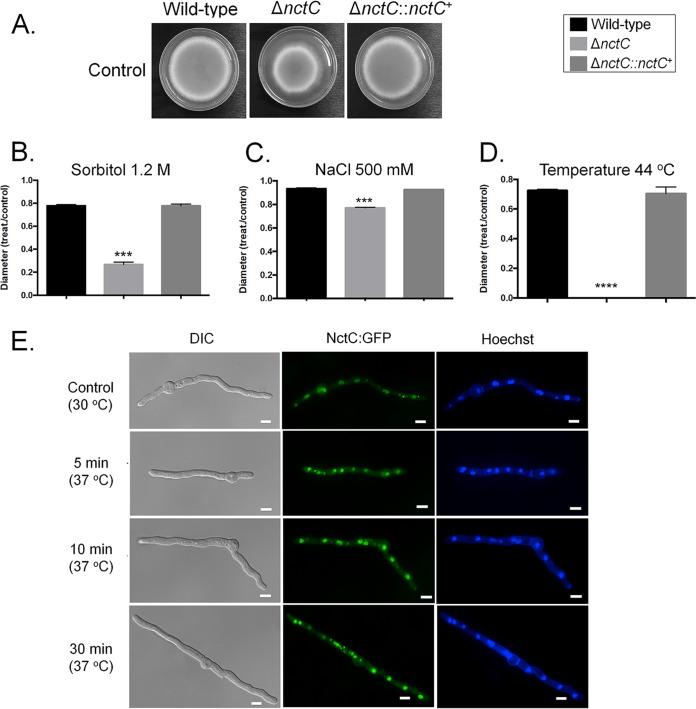
Molecular characterization of NctC. (A to D) Growth phenotypes of the wild-type, Δ*nctC*, and Δ*nctC*::*nctC*^+^ strains grown for 5 days in MM at 37°C (A), MM plus sorbitol 1.2 M (B), MM plus NaCl 500 mM (C), and MM at 44°C (D). Standard deviations represent averages of results from three independent biological repetitions Statistical analysis was performed using one-tailed, paired *t* tests for comparisons to the control condition (*, *P* < 0.05; **, *P* < 0.01; ***, *P* < 0.001; ****, *P* < 0.0001). (E) NctC:GFP germlings (16 h old) were constitutively present in the nuclei at 30°C and 37°C.

**FIG 5:**
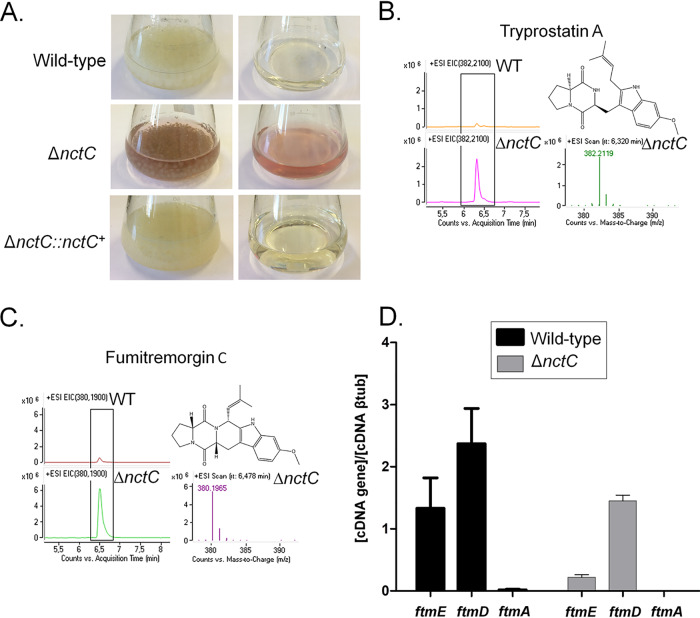
The Δ*nctC* mutant has increased production of several secondary metabolites. (A) Cultures (left column) and supernatants separated from the same cultures (right column) were grown with agitation for 5 days at 37°C. Secondary metabolites were extracted from the wild-type and Δ*nctC* cultures from those supernatants, and mass spectrometry was performed, identifying tryprostatin A (B) and fumitremorgin C (C). (E) Determination of the expression of *ftmE*, *ftmD*, and *ftmA* by RT-qPCR. The wild-type and Δ*nctC* strains were grown for 48 h at 37°C. Gene expression was normalized using *tubA* (Afu1g10910). Standard deviations represent averages of results from three independent biological repetitions. Statistical analysis was performed using one-way ANOVA for comparisons to the wild-type condition (*, *P* < 0.05).

The Δ*nctC* mutant showed (about 30%) reduced growth in MM compared to the wild-type and complemented strains (Fig. 4A). It also displayed reduced growth in high osmotic concentrations and at a high temperature (44°C) (Fig. 4B to D). NctC:GFP was constitutively present in the nuclei, and its localization was not affected by switching the temperature or exposure to the aforementioned stressing conditions (Fig. 4E and data not shown). Interestingly, the Δ*nctC* mutant also secreted reddish compounds in the culture medium under conditions of growth at 37°C for 48 h (Fig. 5A). Mass spectrometry analysis showed that the Δ*nctC* mutant had increased production of fumitremorgin C and tryprostatin A (Fig. 5B and C). Surprisingly, three genes in the fumitremorgin biosynthesis pathway, *ftmE* (AFUB_086320), *ftmD* (AFUB_086340), and *ftmA* (AFUB_060400), did not shown mRNA accumulation in the Δ*nctC* mutant (Fig. 5D).

**FIG 6:**
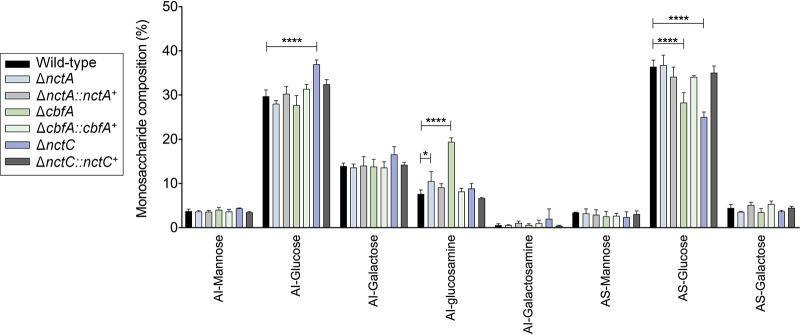
Composition of monosaccharides in the cell walls of the wild-type and mutant strains. Standard deviations represent averages of results from three independent biological repetitions. Statistical analysis was performed using one-way ANOVA for comparisons to the wild-type condition (*, *P* < 0.05).

There are differences in the cell wall composition of these strains (Fig. 6). The Δ*cbfA* and Δ*nctA* strains had higher levels of alkali-insoluble glucosamine (AI-glucosamine) (representing chitin) and lower levels of alkali-soluble glucose (AS-glucose) (indicating α-1,3-glucan content) than the wild-type and complemented strains (Fig. 6). The Δ*nctC* strain had higher levels of AS-glucose whereas the Δ*nctA* strain had lower levels of AS-glucose and of AI-glucose (representative of β-1,3-glucan) than the wild-type and complemented strains (Fig. 6).

In a neutropenic mouse model, the Δ*cbfA* mutant was as virulent as the wild-type strain whereas the Δ*nctC* mutant was avirulent (see Fig. S1A and S1B at doi.org/10.6084/m9.figshare.12315230).

Overall, these results strongly indicate that CbfA and NctC are important regulators of genes that have an impact not only on caspofungin sensitivity and cell wall composition but also on the responses that occur under several stress conditions.

### FhdA, containing a forkhead-associated domain, is involved in stress tolerance.

**FIG 7:**
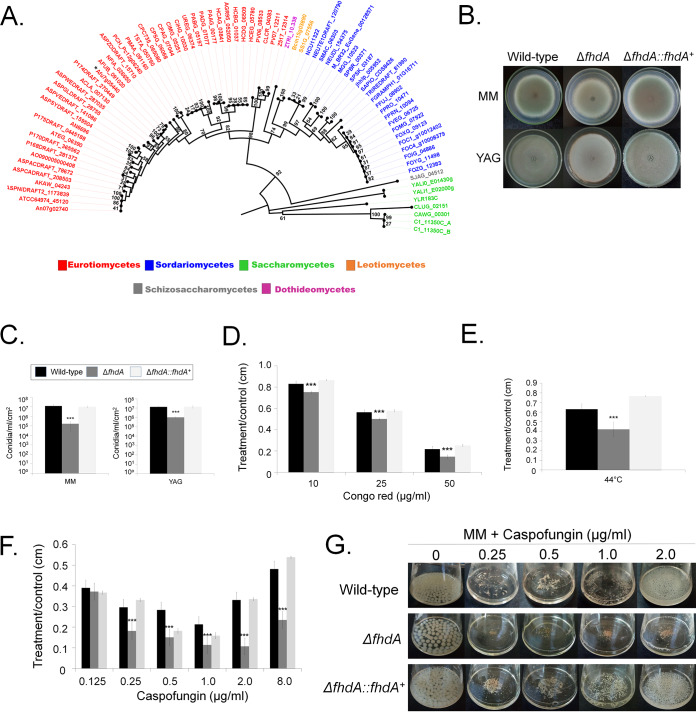
Molecular characterization of FhdA null mutant. (A) Phylogenetic distribution of FhdA across fungal genomes. Sequences were aligned through ClustalW implemented in the MEGA7 software (77). Phylogenetic analyzes were performed using MEGA7 software and the neighbor-joining method (78) and 1,000 bootstrap replications (79) for each analysis. The phylogenetic tree was visualized using the Figtree program (http://tree.bio.ed.ac.uk/software/figtree/). The asterisk (*) indicates FhdA. (B) Growth phenotypes of the wild-type, Δ*fhdA*, and Δ*fhdA*::*fhdA*^+^ strains grown for 5 days at 37°C. (C) Number of conidia in the wild-type, Δ*fhdA*, and Δ*fhdA*::*fhdA*^+^ strains grown for 5 days at 37°C on MM and YAG medium. (D to F) Growth phenotypes on MM plus Congo red grown at 37°C (D) and at the higher temperature of 44°C (E) and on MM plus caspofungin (F). (G) Growth in MM plus caspofungin 2 μg/ml for 48 h at 37°C. Standard deviations represent averages of results from three independent biological repetitions. Statistical analysis was performed using one-tailed, paired *t* tests for comparisons to the control condition (*, *P* < 0.05; **, *P* < 0.01; ***, *P* < 0.001; ****, *P* < 0.0001).

The *fhdA* gene encodes a putative transcription factor of 513 amino acids in length with a molecular weight of 57.1 kDa and a forkhead-associated domain (SM00240; https://smart.embl.de/) (between amino acids 108 and 166). Phylogenetic analysis of FhdA across fungal species representing the 13 different taxonomic classes or subphyla within Dikarya (https://www.fungidb.org) revealed that orthologues were restricted to Pezizomycotina (Eurotiomycetes, Sordariomycetes, Leotiomycetes, and Dothideomycetes), Saccharomycotina (Saccharomycetes), and Taphrinomycotina (Schizosaccharomycetes) (Fig. 7A; see also Table S1 at doi.org/10.6084/m9.figshare.12315230). FhdA orthologues were identified in other important fungal pathogens, such as *Coccidioides* spp., *Fusarium* spp., and Ajellomyces capsulatus (see Table S1).

The FhdA:GFP was constantly present in the nucleus even after caspofungin exposure (about 100 germlings exposed or not to 0.006 μg/ml of caspofungin for 30 min; data not shown). There were no differences in the radial growth among the Δ*fhdA* mutant and wild-type and complemented strains in both MM and YAG medium (2% [wt/vol] glucose, 0.5% [wt/vol] yeast extract, trace elements) (Fig. 7B). However, the Δ*fhdA* mutant showed some conidiation defects since it produced about 10-fold to 100-fold less conidia depending on the culture media (Fig. 7C). The Δ*fhdA* mutant was very sensitive to low caspofungin concentrations and had reduced CPE. Furthermore, it exhibited decreased growth when exposed to Congo red or at higher temperature (Fig. 1, 7D to G). In a neutropenic mouse model, the Δ*fhdA* mutant is as virulent as the wild-type strain (see Fig. S1C at doi.org/10.6084/m9.figshare.12315230). Taken together, these data strongly suggest that FhdA is a TF important for caspofungin, Congo red, and high-temperature tolerance.

### Transcriptional profiling of the Δ*fhdA* mutant after caspofungin exposure.

**FIG 8:**
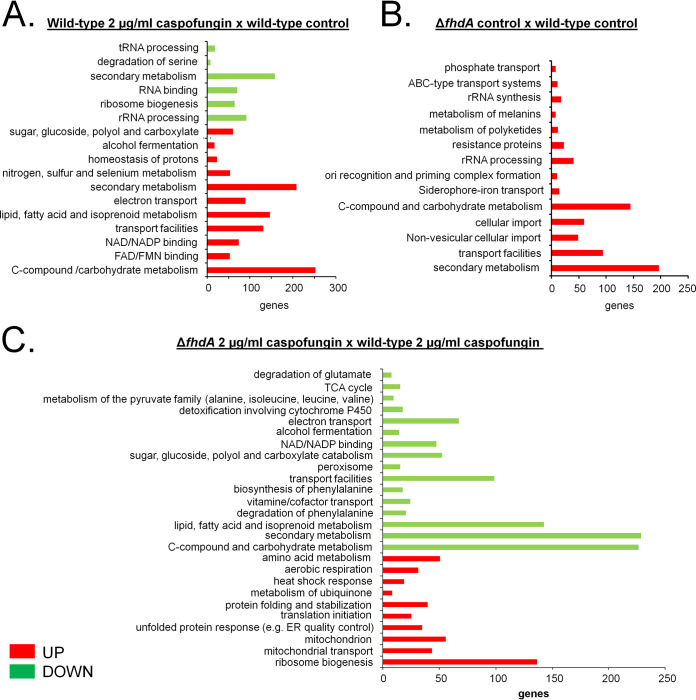
A summary of the FunCat terms overrepresented and upregulated or downregulated (adjusted *P* value, <0.05) upon growth in MM and MM plus caspofungin 2 μg/ml for 48 h at 37°C. (A) Wild-type strain treated with 2 μg/ml caspofungin versus (x) wild-type control. (B) Δ*fhdA* control strain x wild-type control. (C) Δ*fhdA* mutant treated with 2 μg/ml caspofungin x wild-type strain treated with 2 μg/ml caspofungin.

To identify FhdA-dependent gene expression under conditions of extensive exposure to caspofungin, the wild-type and Δ*fdhA* transcriptomes were assessed postexposure to 2 μg/ml caspofungin for 48 h (Fig. 7G). Differentially expressed genes were defined as those with a minimum of 2-fold change (FC) in gene expression (log2FC greater than or equal to 1.0 and less than or equal to −1.0) with a false-discovery rate (FDR) of less than 0.05 (see Table S2 at doi.org/10.6084/m9.figshare.12315230) compared to the wild-type strain under the equivalent conditions. In the wild-type strain, 1,720 and 1,411 genes were upregulated and downregulated, respectively, after 48 h of growth in the presence of caspofungin. FunCat (https://elbe.hki-jena.de/fungifun/fungifun.php) enrichment analyses performed for the wild-type strain demonstrated a transcriptional upregulation of genes coding for proteins involved in C-compound and carbohydrate metabolism; sugar, glucoside, polyol, and carboxylate catabolism; lipid, fatty acid, and isoprenoid metabolism; secondary metabolism; flavin adenine dinucleotide/flavin mononucleotide (FAD/FMN) and NAD/NADP binding; and electron transport (Fig. 8A). On the other hand, there was a downregulation of genes coding for proteins involved in rRNA processing, tRNA processing, RNA binding, ribosome biogenesis, secondary metabolism, and degradation of serine (Fig. 8A). This implies that under conditions of extensive exposure to CPE drug concentrations, A. fumigatus upregulates energy production, carbon blocks for carbohydrate and lipid biosynthesis, and secondary metabolism while downregulating RNA metabolism and part of the secondary metabolism.

Comparisons of the Δ*fhdA* mutant to the wild-type strain under basal conditions (without exposure to caspofungin) revealed enrichment of the transcriptional upregulation of genes encoding proteins related to C-compound and carbohydrate metabolism, transport facilities, siderophore-iron transport, and secondary metabolism (Fig. 8B). Under conditions of constant exposure to caspofungin, the Δ*fhdA* mutant showed enrichment of the upregulation of genes related to amino acid metabolism, aerobic respiration, heat shock response, and mitochondrial transport (Fig. 8C); in addition, enrichment was observed for downregulated genes related to the tricarboxylic acid (TCA) cycle; metabolism of pyruvate; electron transport; alcohol fermentation; lipid, fatty acid, and isoprenoid metabolism; secondary metabolism; and C-compound and carbohydrate metabolism. Taken together, the data suggest that the Δ*fhdA* mutant evidenced some defects in the mitochondrial function that probably affected several pathways related to caspofungin tolerance.

### Independent validation that iron depletion impacts the Δ*fhdA* mutant upon CPE.

**FIG 9:**
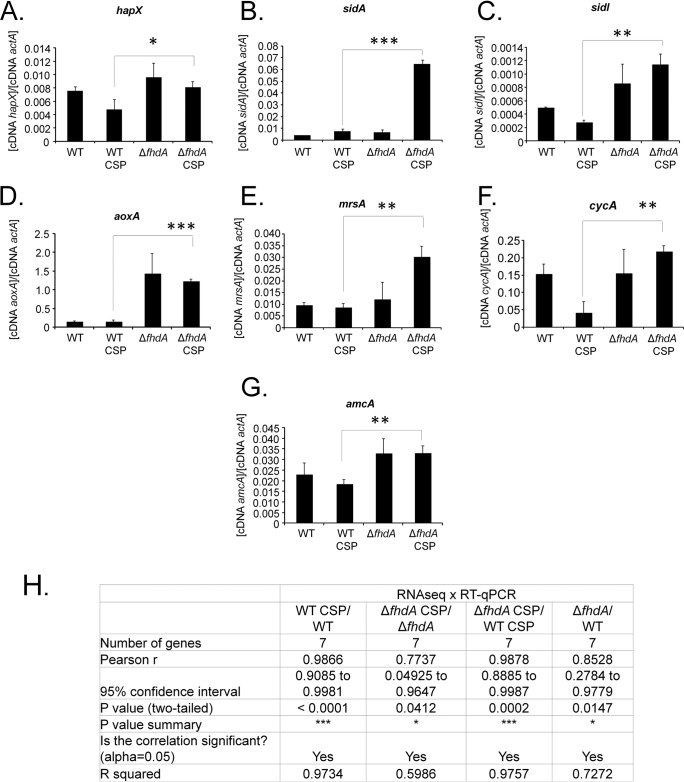
Independent validation of the RNA-seq results. The wild-type and Δ*fhdA* strains were grown for 48 h at 37°C in the presence or absence of caspofungin 2 μg/ml. Gene expression data were obtained for *hapX* (A), *sidA* (B), *sidI* (C), *aoxA* (D), *mrsA* (E), *cycA* (F), and *amcA* (G) and were normalized by using actA (Afu6g04740). Standard deviations represent averages of results from three independent biological repetitions (each with 2 technical repetitions). Statistical analysis was performed using one-way ANOVA for comparisons to the wild-type condition (*, *P* < 0.05). (H) The expression levels of these seven genes measured by RT-qPCR showed a high degree of correlation with the RNA-seq data (Pearson correlation ranging from 0.77 to 0.98).

RT-qPCR experiments validated the transcriptome sequencing (RNA-seq) results determined for all seven genes selected from the caspofungin stress response experiments (Fig. 9A to G). We selected genes involved in iron starvation, such as *hapX* (AFUB_052420), which encodes a bZIP transcription factor required for adaptation to both iron depletion and excess and for transcriptional activation of the siderophore system (33); *sidA* (AFUB_023720), encoding an l-ornithine N5-oxygenase, representing the first committed step in siderophore biosynthesis (34); *sidI* (AFUB_016580), encoding a putative long-chain-fatty-acid–coenzyme A (CoA) ligase, mevalonyl-CoA ligase (35); *amcA* (AFUB_083830), encoding a putative mitochondrial ornithine carrier protein whose expression was upregulated under low-iron conditions (36); *mrsA* (AFUB_078550), which encodes a mitochondrial iron transporter (37); *cycA* (AFUB_028740), encoding a cytochrome c whose transcript was derepressed during iron starvation in a hapX mutant (37); and *aoxA* (AFUB_022090), encoding an alternative oxidase that mediates the activity of the cyanide-insensitive respiratory pathway that was transcriptionally induced in response to oxidative stress (38) (Fig. 9A to G). The *actA* A. fumigatus 6g04740 (Afu6g04740) gene, which codes for α-actin, was used as a normalizer due to its consistent expression in all strains during caspofungin stress (see Table S3 at doi.org/10.6084/m9.figshare.12315230). The expression levels of these seven genes showed a high degree of correlation with the RNA-seq data (Pearson correlation values ranging from 0.7737 to 0.9878; Fig. 9H).

**FIG 10:**
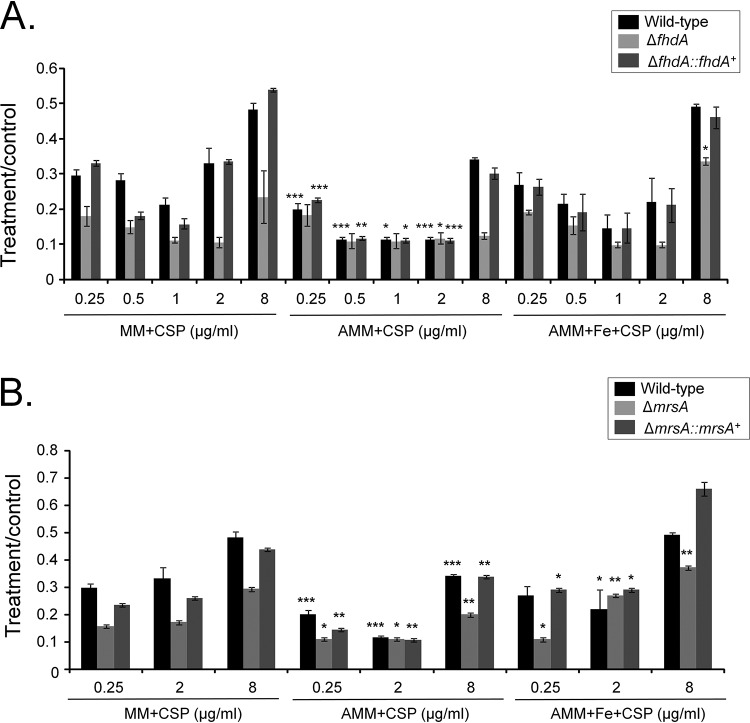
Iron is important for caspofungin resistance and CPE. Growth phenotypes were determined for the wild-type, Δ*fhdA* (A), and Δ*mrsA* (B) strains and the corresponding complementing strains grown for 5 days in MM, AMM, and AMM plus FeSO_4_ 200 μM at 37°C with and without CSP. Standard deviations represent averages of results from three independent biological repetitions. Statistical analysis was performed using one-tailed, paired *t* tests for comparisons to the control condition, MM plus CSP (*, *P* < 0.05; **, *P* < 0.01; ***, *P* < 0.001; ****, *P* < 0.0001). CSP, caspofungin; Fe, FeSO_4_.

Iron is important for resistance to caspofungin and CPE for the wild-type and Δ*fhdA*::*fhdA*^+^ strains since they grew proportionally less extensively in iron-depleted MM (AMM) than in non-iron-depleted MM (MM plus caspofungin [CSP]; Fig. 10A, compare the middle graph to the left graph). Comparisons of growth in AMM and MM showed that there were no growth differences for the Δ*fhdA* mutant (Fig. 10A, compare the middle graph to the left graph). In contrast, iron excess in MM (MM plus Fe) improved Δ*fhdA* growth at lower caspofungin and CPE concentrations, while there were no differences for the wild-type and complementing strains (Fig. 10A, compare the right graph to the left graph).

The Δ*mrsA* strain (*mrsA* encodes a mitochondrial iron transporter [39]) was more sensitive to 0.25 and 2.0 μg/ml of caspofungin in MM and 0.25 μg/ml of caspofungin in AMM than the wild-type and complementing strains (Fig. 10B). It also had a lower CPE in both MM and AMM (Fig. 10B). Iron excess in MM did not improve the growth of the Δ*mrsA* mutant in medium containing 0.25 μg/ml of caspofungin; in contrast, it did improve the growth in medium containing 2 and 8 μg/ml of caspofungin (Fig. 10B).

Taken together, these data indicate that FhdA connects iron repletion to caspofungin resistance and CPE.

### FhdA is important for the mitochondrial respiratory function.

The RNA-seq analysis suggested that FhdA was also important for the mitochondrial function upon CPE (Fig. 8). Thus, we verified the state of mitochondria in living cells by using Mitotracker green as a mitochondrial fluorescent probe. When A. fumigatus wild-type and Δ*fhdA* germlings were left unexposed to caspofungin, they displayed an intact mitochondrial network. In contrast, when germlings of both the strains were exposed to caspofungin, they showed a mitochondrial network fragmentation process, revealed by an intense punctuated fluorescent distribution in the cytoplasm, in 100% and 60% of the germlings, respectively.

**FIG 11:**
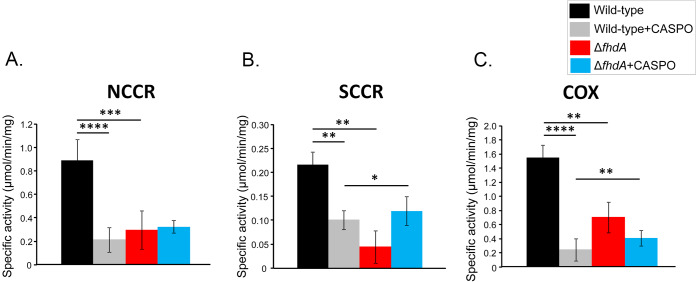
FhdA is important for mitochondrial respiratory function in A. fumigatus. Enzymatic activities were determined for NADH cytochrome c reductase (NCCR, representing complexes I and III) (A), succinate-cytochrome c reductase (SCRR, representing complexes II and III) (B), and cytochrome c oxidase (COX, representing complex IV) (C). Statistical analysis was performed using one-tailed, paired *t* tests for comparisons to the control condition (*, *P* < 0.05; **, *P* < 0.01; ***, *P* < 0.001; ****, *P* < 0.0001).

Alternatively, we subjected cellular extracts from wild-type and Δ*fhdA* strains to mitochondrial fractionation and evaluated their respiratory competence by measuring the activities of mitochondrial complexes I to IV (Fig. 11; see also Fig. S2 at doi.org/10.6084/m9.figshare.12315230). NADH cytochrome c reductase (NCCR, representing complexes I and III), succinate-cytochrome c reductase (SCRR, representing complexes II and III), and cytochrome c oxidase (COX, representing complex IV) exhibited 4-fold, 2-fold, and 7-fold lower activities in the Δ*fhdA* mutant than in the wild-type strain, respectively (Fig. 11). The wild-type NCCR, SCCR, and COX activities were also reduced about 2-fold to 7-fold when the strain was exposed for 48 h to caspofungin 2 μg/ml (Fig. 11). The decrement in the activity of the tested respiratory complexes of caspofungin-treated wild-type cells is in agreement with the observed organellar fragmentation. In contrast, the NCCR, SCCR, and COX activities were the same or were increased or reduced about 2-fold under conditions of exposure of the Δ*fhdA* mutant to caspofungin 2 μg/ml for 48 h (Fig. 11). In this case, the fragmentation of the mitochondrial network did not result in an additive effect in the respiratory impairment of the Δ*fhdA* cells. Mitochondrial network fragmentation is frequently associated with the organellar quality control and mitophagy (40); therefore, the removal of damaged mitochondria can provide some compensation for the already prejudiced respiration observed in Δ*fhdA* cells.

## DISCUSSION

The echinocandin caspofungin, considered a second-line therapy for use in combatting IA, is a β-1,3-glucan synthase inhibitor and, when used in high concentrations, restores the anticipated A. fumigatus growth inhibition, a phenomenon called the “caspofungin paradoxical effect” (CPE). CPE has been widely associated with increased chitin content in the cell wall due to a compensatory upregulation of chitin synthase-encoding genes (16, 41). We have previously demonstrated that CPE depends on the cell wall integrity (CWI) mitogen-activated protein kinase MpkA and its associated TF RlmA, which regulates chitin synthase gene expression in response to different concentrations of caspofungin (23, 29). Moreover, we found that the calcium- and calcineurin-dependent TF CrzA binds to and regulates the expression of specific chitin synthase genes in the absence of caspofungin and under CPE conditions (23). We also identified a novel basic leucine zipper TF, ZipD, that is also involved in CPE. Here, we have identified 11 TFs that are involved in the A. fumigatus CPE. Among these TFs, nine are important not only for caspofungin tolerance but also for resistance. Six of them (Δ*cbfA*, Δ*zipD*, Δ*nctC*, Δ*znfA*, Δ*atfA*, and Δ*rlmA*) have been previously identified as being associated with calcium sensitivity and caspofungin sensitivity and displaying reduced CPE (22, 23, 28, 42). ZipD and RlmA are important for osmotic and cell wall stresses, collaborating for cell wall construction and the activation of several pathways important for osmotic stability (22, 23). Here, we demonstrated that there were increased levels of chitin composition in the cell wall of the Δ*cbfA* and Δ*nctA* mutants but not in that of the Δ*nctC* mutant. We previously demonstrated that the Δ*zipD* and Δ*rlmA* mutants have altered chitin composition (22, 23). It remains to be determined if the chitin concentration is altered in the Δ*nctB*, Δ*fhdA*, Δ*znfA*, Δ*atfA*, Δ*zfpA*, and ΔAFUB_054000 mutants. The identification of these TFs increases the number of genetic determinants known for caspofungin resistance and CPE and provides new opportunities for the identification of signaling pathways important for caspofungin resistance and tolerance.

NctA, NctB, NctC, and CbfA are members of the evolutionarily conserved CBF/NF-Y family of transcription factors (26). Δ*cbfA* not only was more sensitive to caspofungin and displayed reduced CPE but also was more sensitive to osmotic, cell wall, high temperature, and calcium stresses. CbfA was constantly present in the nucleus, and its localization was not affected by different stimuli. Interestingly, derepression of several secondary metabolites was seen in the Δ*cbfA* mutant, suggesting that CbfA is important for the regulation of the biosynthesis of A. fumigatus secondary metabolites. CbfA is the homologue of Dpb4p, one of the subunits of the S. cerevisiae DNA polymerase epsilon (43). Loss of the DPB4 gene in S. cerevisiae is not lethal but disturbs DNA replication (43). On the other hand, a C. albicans Dpb4p homologue has been identified as one of the regulators of Goa1p, a protein required for respiratory growth and resistance to oxidative agents (44, 45). C. albicans Δdpb4 is more sensitive to cell wall inhibitors, including caspofungin, and shows defects in mitochondrial function (45).

NctA (AFUB_029870) is the putative homologue of the S. cerevisiae negative cofactor 2 (NC2) complex α-subunit Bur6p. NctA has two paralogues in A. fumigatus, NctC and HapE. In addition, NctB is a putative homologue of the S. cerevisiae NC2 complex β-subunit Ncb2p. It has been shown that, as in the case of S. cerevisiae (Bur6p and Ncb2p), A. fumigatus NctA and NctB physically interact (25). These two TFs are subunits of the heterotrimeric NC2 complex that acts as a negative regulator of RNA polymerase II transcription by inhibiting formation of the preinitiation complex (46, 47). Genome-wide studies in S. cerevisiae, C. albicans, A. fumigatus, and Homo sapiens have shown that the N-chlorotaurine (NCT) complex interacts with 20% to 30% of all RNA Pol II gene promoters (25, 48–51).

Similarly to the Δ*cbfA* mutant, the Δ*nctC* mutant not only was more sensitive to caspofungin and exhibited a reduced CPE but also was more sensitive to several different stresses, such as osmotic stress and higher temperature. NctC was also constantly present in the nucleus, and its localization was not affected by different stimuli. In this mutant, derepression of several secondary metabolites was also observed, suggesting that not only CbfA but also NctC is important for the regulation of the synthesis of secondary metabolites in A. fumigatus. Recently, A. fumigatus *nctA* and *nctB* null mutants were shown to phenocopy each other, being more resistant to itraconazole, voriconazole, posaconazole, terbinafine, miltefosine, and amphotericin and more sensitive to cell wall-perturbing agents such as Congo red, calcofluor white, caspofungin, and micafungin (25). We were not able to observe caspofungin sensitivity in the Δ*nctA* mutant or resistance to azoles in the Δ*nctB* mutant, which could have been due to the different conditions used for the MIC and growth assays. It was reported previously that, in azole-resistant isolates of C. albicans, the azole transporter CDR1, which is regulated by the Ncb2 homologue, was overexpressed (52). Conditional NCB2 null mutants displayed decreased susceptibility toward azole drugs and enhanced transcription of CDR1 (52).

Four TFs, ZnfA, FhdA, ZfpA, and AFUB_054000, are completely novel and have not yet been characterized. In addition to its sensitivity to caspofungin and reduced CPE, the Δ*znfA* mutant is more sensitive to calcium than the wild-type strain (22). The ZfpA mutant has increased mRNA accumulation in the presence of voriconazole or calcium (31, 32, 53). We investigated in more detail the FhdA TF; the Δ*fhdA* mutant not only was sensitive to caspofungin and had reduced CPE but also was more sensitive to Congo red exposure and high temperature. RNA-seq experiments performed with the Δ*fhdA* mutant in the absence or presence of caspofungin suggested that this strain had some defects in the mitochondrial function. Mitochondrial pathways such as those associated with amino acid degradation, electron transport chains, some mitochondrial lipids, fatty acids, and isoprenoid metabolism were downregulated when the Δ*fhdA* mutant was exposed to caspofungin in comparison with the wild-type strain. The Δ*fhdA* mutant had reduced mitochondrial fragmentation and lower levels of mitochondrial enzymatic activities, indicating its involvement in the functioning of mitochondria. The connection between mitochondrial function and caspofungin tolerance has been widely reported in the literature (15, 39, 54–58). Addition of respiratory inhibitors results in significant decreases of the caspofungin MICs for C. parapsilosis (39). Caspofungin exposure induces expression of A. fumigatus mitochondrial respiratory chain genes, specifically NADH-ubiquinone oxidoreductases (complex I), while CPE is abolished by addition of rotenone, a complex I inhibitor (58). Interestingly, those authors also described a connection between rotenone inhibition and increases in intracellular calcium concentrations. Recently, it has been reported that caspofungin induced mitochondrion-derived reactive oxygen species (ROS) and that inhibition of ROS formation eliminated caspofungin-induced resistance (15). Those authors proposed a novel mechanism of caspofungin resistance through ROS production, as well as integrated oxidative stress and sphingolipid alterations (15). We have extended those studies by demonstrating that caspofungin induced mitochondrial fragmentation and inhibited all the tested respiratory complexes in the wild-type strain.

A link between iron chelation and caspofungin susceptibility has also been observed (59, 60). Gallium is a metal compound that can be taken up by cellular iron transport systems, replacing it in iron-containing proteins. However, it cannot be reduced under physiological conditions, inhibiting the functionality of gallium-complexed proteins and arresting growth (59). We demonstrated previously that gallium nitrate III [Ga(NO_3_)_3_] has a synergistic effect with caspofungin but not with azoles and that it impairs CPE (59). We have observed several genes that encode proteins important for iron assimilation were more highly expressed in the Δ*fhdA* mutant, suggesting problems involving iron deficiency in this mutant. Mitochondria employ the majority of cellular iron and therefore have a central role in its homeostasis. Actually, addition of iron together with caspofungin improved the CPE in the Δ*fhdA* mutant whereas iron depletion in the presence of caspofungin increased caspofungin sensitivity and decreased CPE. Interestingly, the same effect was observed in the Δ*mrsA* mutant (*mrsA* encodes a mitochondrial iron transporter), suggesting that iron deficiency in mitochondria affects caspofungin sensitivity and CPE.

Our work opens new possibilities for understanding caspofungin tolerance and resistance in fungi. Although the clinical significance of CPE remains unclear (16, 61), those pathways that are modulated during caspofungin responses could be considered to be promising targets for drug development. The implementation of the use of drugs targeting these pathways together with caspofungin could help to improve caspofungin efficacy and, ultimately, aspergillosis treatment.

## MATERIALS AND METHODS

### Strains and media.

Strains were grown at 37°C in either complete medium (YAG medium) (2% [wt/vol] glucose, 0.5% [wt/vol] yeast extract, trace elements) or minimal medium (1% [wt/vol] glucose, nitrate salts, trace elements, pH 6.5). Solid YAG medium and MM were the same as described above except that 1.7% (wt/vol) or 2% (wt/vol) agar was added. Trace elements, vitamins, and nitrate salts compositions were as described previously (62). For iron limitation experiments, strains were growth in solid AMM (containing glucose 1% [wt/vol], trace elements without FeSO_4_, agar 1.7% [wt/vol]). Wherever required, MM was supplemented, at the stated concentrations, with calcium chloride (CaCl_2_), sorbitol, sodium chloride (NaCl), Congo red, iron sulfate (FeSO_4_), and caspofungin.

For phenotype characterization, solid-medium plates were inoculated with 10^4^ spores per strain and the spores were left to grow for 120 h at 37, 30, or 44°C. Values representing levels of radial growth were expressed as ratios, determined by dividing colony radial diameter of growth under the stress condition by colony radial diameter under the control (no stress) condition. All the strains used in this work are described in Table S4 at doi.org/10.6084/m9.figshare.12315230.

### Construction of A. fumigatus mutant strains.

TF deletion mutants of A. fumigatus were described previously (25) and were obtained as described in reference 63. All GFP fusion constructions were performed by in vivo recombination in S. cerevisiae as previously reported (64), and all methods were adapted to A. fumigatus as previously described (65). Approximately 1 kb from the 5′ untranscribed region (5′-UTR)- and 3′-UTR-flanking sequences of the targeted genes were selected for primer design. The 5F and 3R primers contained a short sequence homologous to the multiple-cloning site of the pRS426 plasmid. For NctC:GFP constructions, the *pyrG* gene was used as a marker for prototrophy and was amplified from the pCDA21 plasmid (66). For CbfA:GFP and FhdA:GFP constructions, the GFP-pyrG-trpC fragment was amplified from the pOB435 plasmid. Cassettes used for A. fumigatus transformation were amplified directly from yeast genomic DNA using TaKaRa Ex Taq DNA polymerase (Clontech TaKaRa Bio). MM was used as selective media for positive transformants.

All complemented strains were obtained by cotransformation of the open reading frame (ORF) and plasmid pPTRI containing the gene that confers resistance to pyrithiamine. MM supplemented with pyrithiamine (1 μg/ml) was used as selective media for positive transformants. Southern blotting and PCR analyses were used to verify homologous cassette integration into the A. fumigatus genome (see Fig. S3 at doi.org/10.6084/m9.figshare.12315230). The primers used in this work are listed in Table S5.

### Microscopy.

Conidiospores were grown on coverslips in 4 ml of liquid MM for 16 h at 30°C. After incubation, coverslips with adherent germlings were left untreated or treated with Hoechst 33342 dye (Molecular Probes, Eugene, OR, USA) 20 μg/ml or MitoTracker green FM dye (Molecular Probes) (250 nM) for 10 min. For caspofungin experiments, after overnight incubation, cells were exposed to caspofungin for 1 h (0.003 or 0.006 μg/ml) for 30 min or left unexposed. Subsequently, the coverslips were rinsed with phosphate-buffered saline (PBS; 140 mM NaCl, 2 mM KCl, 10 mM NaHPO_4_, 1.8 mM KH_2_PO_4_, pH 7.4) and mounted for examination. Slides were visualized on an Observer Z1 fluorescence microscope using a 100× objective oil immersion lens objective. For GFP and MitoTracker green conditions, filter set 38 (high efficiency [HE]) was used with an excitation wavelength of 450 to 490 nm and emission wavelength of 500 to 550 nm. For Hoechst/DAPI [4,6-diamidino-2-phenylindole] staining, filter set 49 was used with an excitation wavelength of 365 nm and emission wavelength of 420 nm to 470 nm. DIC (differential interference contrast) images and fluorescent images were captured with an AxioCam camera (Carl Zeiss, Oberkochen, Germany) and processed using AxioVision software (version 4.8).

### RNA extraction and qRT-PCR.

After the stated treatments were performed, mycelia were collected and ground to a fine powder in liquid N_2_. RNA was extracted with using TRIzol reagent (Invitrogen, Life Technologies, Camarillo, CA, USA) following DNA digestion with RQ1 RNase-free DNase (Promega, Fitchburg, WI, USA) according to the manufacturer’s instructions. Total RNA was reverse-transcribed into cDNA by using an ImProm-II reverse transcription system (Promega) and oligo(dT). The amplification assay was carried out in a model 7500 real-time PCR system (Applied Biosystems). qPCRs were performed in a 10-μl final volume containing Sybr green PCR master mix (Applied Biosystems) under the following conditions: an initial step of 2 s at 50°C, followed by 10 min at 95°C and 40 cycles at 95°C for 15 s and 60°C for 1 min. Three independent biological replicates were used, and mRNA quantity relative fold change data were calculated using standard curves (67) and normalized by β-tubulin or α-actin expression. All the primers used for RT-qPCR experiments are described in Table S5 at doi.org/10.6084/m9.figshare.12315230.

### RNA sequencing.

A. fumigatus conidia (5 × 10^7^) from the parental CEA17 and Δ*fhdA* mutant strains were inoculated in triplicate into liquid MM and cultured for 8 h at 37°C prior to the addition or not of 2 μg/ml caspofungin and left to grown for 48 h. Mycelia were harvested, frozen, and ground in liquid nitrogen. Total RNA was extracted using TRIzol (Invitrogen), treated with RQ1 RNase-free DNase I (Promega), and purified using an RNeasy kit (Qiagen) according to manufacturer’s instructions. RNA from each treatment was quantified using a Qubit fluorometer and analyzed using an Agilent 2100 Bioanalyzer system to assess the integrity of the RNA. RNA integrity number (RIN) values were calculated and ranged from 7.0 to 9.5.

An Illumina TruSeq stranded mRNA sample preparation kit was used to construct cDNA libraries following the manufacturer’s instructions. Libraries were sequenced (2 × 100 bp) at the next-generation sequencing (NGS) facility of the Brazilian Bioethanol Science and Technology Laboratory (CTBE) using a HiSeq 2500 instrument, generating approximately 11 × 10^6^ fragments per sample.

Obtained fastq files were quality checked with FastQC (http://www.bioinformatics.babraham.ac.uk/projects/fastqc/) and cleaned (quality trim, adaptor removal, and minimum length filtering) with Trimmomatic (68). rRNA was removed using SortMeRNA (69). High-quality RNA-seq reads were mapped against the A. fumigatus genome using CLC Genomics Workbench software (CLC bio—v4.0; Finlandsgade, Denmark) and the following parameters: for the mapping settings, minimum length fraction = 0.7, minimum similarity fraction = 0.8, and maximum number of hits for read = 1; for the alignment settings, minimum distance = 180 and maximum distance = 1,000. All samples achieved saturation of known exon-exon junctions. Reproducibility among biological replicates was assessed by exploring a principal-component-analysis (PCA) plot of the top 500 genes that had the largest biological variation between the libraries and by pairwise measuring of the Pearson correlations among the replicates over the whole set of genes. In order to assess transcript abundance, exonic reads were counted in a strand-specific way using the featureCounts function from the Rsubread Bioconductor package (70). Calling of differentially expressed genes was carried out using DESeq2 (71) and, as the threshold, an adjusted *P* value of <0.01 (72).

### Extraction of secondary metabolites and high-resolution mass spectrometry (HRMS) analyses.

For mass spectrometry analyses, extractions were performed following a previously described methodology (73) with few modifications. Briefly, 100 mg of the lyophilized fungal supernatant was extracted with 1 ml of MeOH during 1 h in an ultrasonic bath. The extracts were centrifuged at 13,000 rpm for 5 min, and the supernatants were collected and dried under N_2_. Crude extracts were resuspended in 1 ml MeOH and filtered using a 0.22-μm-pore-size filter. All the extractions were performed in triplicate.

The liquid chromatography-HRMS (LC-HRMS) analyses were performed in a LC Agilent 1200 liquid chromatography mass spectrometer coupled to an Agilent iFunnel 6550 quadrupole-time of flight (Q-ToF) LC-MS system with an electrospray ionization (ESI) source. All operations and spectrum analyses were conducted using Agilent Mass Hunter Workstation software. The parameters of MS analysis were set as follows: ESI source in positive mode; nebulizing gas temperature at 290°C; capillary voltage at +3,000 V; nozzle voltage at 320 V; drying gas flow of 12 ml min^−1^; nebulization gas pressure at 45 lb/in^2^; auxiliary gas temperature at 350°C; auxiliary gas flow at 12 ml min^−1^; m/z mass range of 50 to 1,700. The chromatography was performed on a Thermo Scientific Accucore C_18_ column (2.6-μm pore size, 2.1 mm by 100 mm). For the gradient elution, 0.1% formic acid (solvent A) and acetonitrile (solvent B) were used, and the eluent profile (A:B) was as follows: 0 to 10 min, gradient from 95:5 to 2:98; 10 to 15 min and final isocratic elution at 2:98. The flow rate was set at 0.2 ml min^−1^, and a 6-μl volume of sample was injected in each injection.

### Mycelial cell wall analysis.

Conidia (1 × 10^8^) were inoculated into 30 ml liquid MM. After 24 h of growth at 37°C, mycelia were harvested by filtration, washed extensively with and suspended in Milli-Q water, and disrupted using a FastPrep homogenizer (MP Biomedicals) at 4°C with 0.5-mm-diameter glass beads. The mycelial suspension obtained after disruption was centrifuged (5,000 rpm, 10 min at 4°C), and the pellet (cell wall) was washed with Milli-Q water (three times); boiled in a water bath upon suspension of the pellet in Tris-HCl buffer (50 mM; pH 7.5) supplemented with EDTA (50 mM), SDS (2%), and β-mercaptoethanol (β-ME, 40 mM) for 15 min (two times); and washed with Milli-Q water (five times; centrifugation at 5,000 rpm for 10 min). The pellet (cell wall) obtained was subjected to alkali fractionation by incubation of the pellet in NaOH (1 M) containing NaBH_4_ (0.5 M) at 65°C for 1 h (two times). The insoluble fraction (alkali insoluble; AI) obtained after centrifugation of the alkali-fractionated cell wall was washed with Milli-Q water (six times; centrifugation at 5,000 rpm for 10 min) and freeze-dried. Excess of NaBH_4_ in the supernatant (alkali-soluble fraction; AS) was neutralized with acetic acid (2%), dialyzed against water until neutrality was reached, and freeze-dried. The AI and AS fractions were then subjected to gas chromatography (GC)-liquid chromatography (Perichrom, France) using an instrument equipped with a flame ionization detector and a fused silica capillary column of 30 m and 0.32 mm internal diameter filled with BP1. In brief, AI and AS suspensions (100 μg; 10 mg/ml stock), containing 4 μg myo-inositol (internal standard), were hydrolyzed with trifluoroacetic acid (4 N; to determine levels of hexoses) and hydrochloric acid (8 N; to determine levels of hexosamines) at 100°C for 4 h, dried under vacuum using NaOH pellets, reduced with NaBH_4_ (10 mg/hydrolyzed sample in 1 ml of 0.1 M NH_4_OH) for 8 to 10 h, dried under vacuum, and washed with methanol (two times). Acetic anhydride (200 μl) and pyridine (50 μl) were added to each of these samples followed by incubation for 10 to 12 h for derivatization of hydrolyzed monosaccharides into alditol acetates. They were then subjected to chloroform-water fractionation, and the derivatives extracted in the chloroform fractions were dried, suspended in methanol, and subjected to GC analyses. The monosaccharide composition was determined from the GC peak area in comparison with the peak area of the internal standard (myo-inositol). The analysis was performed with three biological replicates.

### Cell fractionation and mitochondria isolation.

Mitochondria were isolated from A. fumigatus mycelium (10 g moist mass) based on a previously described protocol (74). Wild-type and Δ*fhdA* conidia (1 × 10^8^) were precultured on 100 ml of MM for 8 h at 37°C prior to addition of 2 μg/ml caspofungin and left to grown for 48 h to induce cell wall stress. The control was left untreated. Mycelia were harvested by filtration in Miracloth (Calbiochem), frozen in liquid nitrogen, and subjected to cell fractionation methods. Mitochondrial extracts were quantified by the use of a modified Lowry method (75) and loaded on 12% SDS-PAGE, electroblotted to PVDF (polyvinylidene difluoride) membranes (GE Health Care), and subsequently analyzed by Western blotting. Monoclonal VDAC1/porin antibody (16G9E6BC4) from S. cerevisiae (Thermo Scientific) and polyclonal homemade anti-CytC antibody (a gift from Mário H. Barros) were used to confirm mitochondrial membrane enrichment.

### Mitochondrial enzymatic activities.

Mitochondrial succinate-cytochrome c reductase (SCCR), NADH-cytochrome c reductase (NCCR), and cytochrome c oxidase (COX) were all assayed spectrophotometrically at 23°C, as previously described (76). Briefly, NCCR and SCCR activities were measured by following the increase in absorbance at 550 nm due to the reduction of cytochrome c in the presence of 10 mM NADH (NCCR) or 10 mM succinate (SCCR). COX was assayed by following the oxidation of ferro-cytochrome c at 550 nM. The specific activity was calculated using an extinction coefficient of 18 mM^−1^ cm^−1^ for cytochrome c.

### Murine model of pulmonary aspergillosis.

Outbred female BALB/c mice (body weight, 20 to 22 g) were housed in vented cages containing 5 animals. Mice were immunosuppressed with cyclophosphamide (150 mg/kg of body weight) administered intraperitoneally on days −4 and −1 prior to infection and day 2 postinfection. Hydrocortisone acetate (200 mg/kg of body weight) was injected subcutaneously on day −3. A. fumigatus conidia grown in YAG plates for 2 days prior to infection were harvested in PBS and filtered through a Miracloth (Calbiochem). Conidial suspensions were washed three times with PBS, counted using a hemocytometer, and resuspended at a concentration of 5 × 10^6^ conidia/ml. The viability of the administered inoculum was determined by incubating a serial dilution of the conidia on YAG medium, at 37°C. Mice were anesthetized by halothane inhalation and infected by intranasal instillation of 10^5^ conidia in 20 μl of PBS. As a negative control, a group of five mice received PBS only. Mice were weighed every 24 h from the day of infection and visually inspected twice daily. The experiments were repeated at least twice, and 10 animals were used for each strain. The statistical significance of the comparative survival values was calculated using log rank analysis and the Prism statistical analysis package (GraphPad Software Inc.).

The principles that guide our studies are based on the Declaration of Animal Rights ratified by UNESCO 27 January 1978 (articles 8 and 14). All protocols adopted in this study were approved by the local ethics committee for animal experiments of the University of São Paulo, Campus of Ribeirão Preto (permit 08.1.1277.53.6; studies on the interaction of Aspergillus fumigatus with animals). Groups of five animals were housed in individually ventilated cages and were cared for in strict accordance with the principles outlined by the Brazilian College of Animal Experimentation (COBEA) and in the Guiding Principles for Research Involving Animals and Human Beings, American Physiological Society. All efforts were made to minimize suffering. Animals were clinically monitored at least twice daily and humanely sacrificed if moribund (defined by lethargy, dyspnea, hypothermia, and weight loss). All stressed animals were sacrificed by cervical dislocation.

### Antifungal susceptibility testing.

The MIC of azole drugs (voriconazole, itraconazole, and posaconazole; Sigma-Aldrich, St. Louis, MO, USA) and amphotericin B (Sigma-Aldrich) was determined using increasing concentrations of the aforementioned compounds in RPMI 1640 based on protocol M38 (2017) of the antifungal microdilution test proposed by the Clinical and Laboratory Standards Institute (CLSI) (https://clsi.org/media/1711/clsistandardsdevelopmentpoliciesandprocessesfinal.pdf).

### Statistical analysis.

Grouped column plots with standard deviation error bars were used for representations of data. For comparisons with data from wild-type or control conditions, we performed one-tailed, paired *t* tests (Fig. 1, 2, 4, 7, 10, and 11) and one-way analysis of variance (ANOVA) (Fig. 3, 5, 6, and 9). *P* values of <0.05 were considered significant. *P* values are indicated by asterisks as follows: *, *P* ≤ 0.05; **, *P* ≤ 0.01; ***, *P* ≤ 0.001; ****, *P* ≤ 0.0001. Graphs were constructed and *t* tests performed by using 2010 Excel 14.0 software (Microsoft Office, 2011), while each ANOVA was performed by using GraphPad Prism 5.00 (GraphPad Software).

### Data availability.

Short reads were submitted to the NCBI’s Short Read Archive under BioProject accession no. PRJNA622251.
